# Mesenchymal stem cells establish a pro-regenerative immune milieu after decellularized rat uterus tissue transplantation

**DOI:** 10.1177/20417314221118858

**Published:** 2022-08-20

**Authors:** Edina Sehic, Emy Thorén, Ingigerdur Gudmundsdottir, Mihai Oltean, Mats Brännström, Mats Hellström

**Affiliations:** 1 Laboratory for Transplantation and Regenerative Medicine, Sahlgrenska Academy, University of Gothenburg, Gothenburg, Sweden; 2 Department of Obstetrics and Gynecology, Clinical Sciences, Sahlgrenska Academy, University of Gothenburg, Gothenburg, Sweden; 3 Department of Surgery, Clinical Sciences, Sahlgrenska Academy, University of Gothenburg, Gothenburg, Sweden; 4 Stockholm IVF-EUGIN, Stockholm, Sweden

**Keywords:** Decellularization, immune response, recellularization, regenerative medicine, transplantation

## Abstract

Decellularized tissue is generally considered immune privileged after transplantation and is an attractive scaffold type for tissue regeneration, including applications for infertility treatment. However, the immune response following transplantation of decellularized tissue is insufficiently studied, in particular after they have been recellularized with mesenchymal stem cells (MSCs). Therefore, we replaced a large uterus segment with a bioengineered graft developed from decellularized uterus tissue and analyzed the immune response during the first 4 months in acellular or MSCs-recellularized scaffolds in the rat. Immunohistochemistry-stained infiltrating immune cells and plasma levels for 16 cytokines and chemokines were quantified. Results revealed that MSCs created an advantageous microenvironment by increasing anti-inflammatory interleukin 10 levels, and increasing the population of FOXP3^+^ T_Regs_ and CD163^+^ M2 macrophages, and by reducing the CD8^+^ cytotoxic T cell population. Hence, MSCs should be considered an immunotherapeutic cell source with the ability to dictate regeneration success after decellularized tissue transplantation.

## Introduction

Bioengineering and the creation of personalized donor tissues from biomaterials and cells for transplantation have been studied in numerous organs and animal models. A tissue-specific scaffold can be manufactured in a process called “decellularization” where most immunogenic cellular compartments are removed from the donor tissue while the extracellular matrix (ECM) composition is preserved in a tissue-specific manner. Patent vascular conduits are also preserved, which enables scaffold implantation by true transplantation with vascular anastomoses and immediate blood flow.^1–3^ Importantly, such scaffolds also remain bioactive and can promote angiogenesis and tissue regenerative pathways, despite previous exposure to detergents and other decellularization chemicals.^4^ It is generally considered that the close homology between ECM molecules across species make these scaffolds less immunogenic after transplantation.^5^ Therefore, decellularized tissue is an interesting scaffold type to stimulate organ and tissue regeneration, including as a novel grafting material to treat reproductive disorders. Several groups investigated if decellularized uterus tissue could be used to treat various forms of uterine factor infertility in rodent models.^6–11^ These studies used a partial uterus repair model that mimics a clinical situation where it would be necessary to reduce/replace uterine scars or other defects caused by common uterine surgery procedures (e.g., myomectomy, adenomyomectomy or resection of placental tumors). Results from these studies collectively showed that it is possible to regenerate a significant part of the uterine wall and to restore fertility in rodent models using a scaffold derived from decellularized tissue. Hence, a bioengineered uterus patch may be a clinically relevant treatment option to cure surgery-induced infertility and to reduce the life-threatening risk of uterine rupture during pregnancy. These principles are therefore also being evaluated in larger animal models with the long-term goal of creating a whole bioengineered uterus that could replace a donor in a uterus transplantation setting.^12^

However, even if decellularized tissue is considered immune privileged, very few studies have investigated the immune response after transplantation of decellularized tissue. The complex interactions between different immune cells and various key signals within the immune system play central roles in determining tissue regeneration or tissue destruction.^13–15^ These important details need to be clarified to allow for a smooth translation of bioengineering principles from animal research into the clinic setting. This is of special importance since recent reports suggest that fragmented ECM components and nucleotide residues within the scaffold, caused by the chemicals used in the decellularization process, can act as allo-independent immunoreactive damage associated molecular patterns (DAMPs). Especially when aggressive decellularization detergents are used.^16,17^ However, the immune response may be modulated by the cells used for the recellularization phase of the decellularized tissue. Mesenchymal stem cells (MSCs) are frequently used together with biological scaffolds because they are relatively easy to isolate, are multipotent and have been approved as a cell source for several clinical trials.^18^ Moreover, because of their known immunomodulatory effects and paracrine functions, MSCs is an attractive cell source to combine with decellularized tissue, including for uterus regeneration applications. Yet, the immune response following transplantation of decellularized tissue is insufficiently studied, in particular when combined with MSCs. In the present study we replaced a large uterus segment in a rat model with a bioengineered graft developed from decellularized rat uterus tissue, with or without MSCs, to extensively analyze the subsequent immune modulation for up to 4 months after transplantation.

## Methods

### Animals and study groups

A total of 57 female rats aged 10–12 weeks at the start of the experiment were used in the study. Twenty Sprague Dawley rats (SD; Charles River, Sulzfelt, Germany) were used as donors for the uterus scaffolds generation and 37 SD rats were used as recipients. The recipient rats were randomly assigned into two groups; Group 1 obtained decellularized uterus tissue that were recellularized with MSCs (*n* = 19), and Group 2 obtained decellularized uterus tissue without any added cells (*n* = 18). All animal work had been reviewed and approved by the animal ethics committee in Gothenburg, Sweden under the document 2228/2019.

### Uterus isolation and decellularization

**Figure 1.**
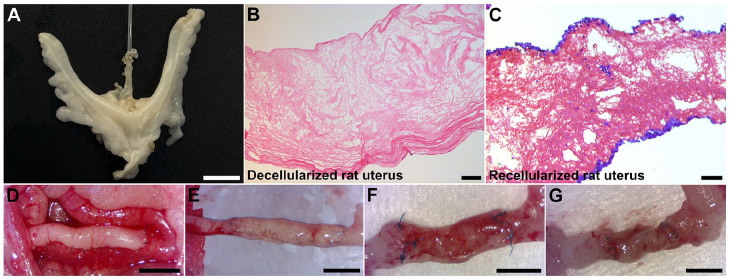
Rat uterus decellularization was conducted through vascular perfusion with DMSO, Triton X-100, and H_2_O, sequentially (A). After a 5-day protocol, the rat uterus became decellularized (B). Decellularized uterus tissue was then recellularized with mesenchymal stem cells that after 2 weeks in vitro had predominantly localized to the superficial layers and in isolated pockets near the injection sites (C; hematoxylin labeled blue cells). Animals received a 20 mm × 10 mm large graft that consisted of decellularized uterus tissue with or without with mesenchymal stem cells (D). Grafts were then assessed at 14 days (E), 30 days (F), and 120 days after engraftment (G). Scale bars; A, 1 cm; B, 100 µm; C, 50 µm; D–G, 5 mm.

Donor uterus used for the whole organ perfusion decellularization process was carefully explanted as described earlier.^8,19^ Briefly, the uterus was isolated with an intact vasculature from uterine arteries, via iliac arteries, to the aorta on the arterial side and from uterine veins, via iliac veins, into the vena cava. The final uterus specimen was then cannulated through the aorta and perfused with a heparinized (50 IU/ml, Leo pharma, Copenhagen, Denmark) phosphate buffer saline solution (PBS; Thermo Fisher Scientific, Gothenburg, Sweden) supplemented with xylocaine (0.4 mg/ml, Astra Zeneca, Gothenburg, Sweden) until the specimen was well blanched. Each isolated uterus was frozen (−20°C) in the perfusate until being thawed for the decellularization process. The decellularization was conducted by vascular perfusion (flow speed = 6 ml/minute) using dimethyl sulfoxide (4% DMSO; Sigma Aldrich, Gothenburg, Sweden) for 4 h, followed by Triton X-100 (1%; Sigma Aldrich) perfusion for 4 h. The perfusion was then continued with deionized water (dH_2_O) for 16 h. This perfusion cycle was then repeated four more times for a total of 5 days. A sterilization step was then performed by perfusing per-acetic acid (0.1%; purity 38%–40%, Merck KGaA, Darmstadt, Germany) for 1 h, followed by a significant washing step using sterile PBS for several hours. This decellularization method has routinely resulted in acellular uterus tissue scaffolds with preserved ECM morphology and with donor DNA levels of less than 35 ng/mg wet tissue, representing <1% of the original DNA content^8,9,16,17^ (Figure 1(A) and (B)). The decellularized uterus was then frozen in sterile PBS at −20°C until further used.

### Scaffold preparation and recellularization

The decellularized uterus was thawed in room temperature and the surrounding adipose tissue and vasculature were removed from each organ. Each uterine horn was then opened longitudinally and trimmed to create a scaffold piece of 20 mm × 10 mm in size. All scaffolds (*n* = 37) were then enzymatically treated to increase the scaffold porosity and potentiate the recellularization efficiency by following a previously established scaffold preconditioning protocol.^4^ Briefly, matrix metalloproteinase (MMP)-2 (2.5 μg/l Peprotech, Stockholm, Sweden) and MMP-9 (2.5 μg/l, Sigma-Aldrich) were prepared using amino-phenylmercuric acetate buffer according to the manufacturer’s instructions. Each scaffold was fully submerged and incubated for 24 h at 37°C in the MMPs solution. The enzyme treated scaffolds were then washed in 20 mM EDTA (Sigma-Aldrich) twice for 20 min followed by several washes in sterile PBS for 1 h. Eighteen acellular grafts were placed in Leibovitz L-15 medium (Merck) and kept at 37°C for up to 6 h before being transplanted. The 19 remaining scaffolds were recellularized with 20 million SD rat bone marrow-derived green fluorescent protein (GFP)-labeled MSCs, respectively (passage number <7; Cyagene Bioscience, cat no. RASMX-01101, Santa Clara, USA), by multiple injections into each scaffold using a 30 G needle and 1 ml syringe. The recellularized scaffolds were submerged in cell culture medium (DMEM glutamax supplemented with 10% FBS and 1% Anti-Anti, Thermo Fisher Scientific) and incubated individually for 2 weeks with trans-well inserts in 6 well-plates (Sarstedt, Nümbrecht, Germany) in standard cell culturing conditions. Culture medium was changed every second day.

### Transplantation of bioengineered construct

Under isoflurane anesthesia, each rat (*n* = 37) was subjected to a laparotomy that exposed the left uterine horn. A 20 mm × 10 mm full-thickness antemesometrial uterus wall segment was excised from the uterus horn leaving the blood vessel filled uterus mesometrium intact. The segment was replaced by an enzyme treated recellularized scaffold (*n* = 19; Group 1; Figure 1(C)) or with an enzyme treated acellular scaffold (*n* = 18; Group 2). Each graft was sutured using 6-0 non-absorbable polypropylene (Ethicon, Raritan, USA) by continuous sutures along the mesodermal side, while interrupted sutures were used for the proximal and distal anastomosis sites (Figure 1(D)). The abdominal muscle layer was then closed with 4-0 silk sutures (Ethicon) and the skin with titanium clips (Reflex7, Gaithersburg, USA). All animals received analgesics and antibiotics post-surgically (buprenorphine 0.05 mg/kg; carprofen 5 mg/kg; sulfamethoxazole 100 mg/kg; trimethoprim 20 mg/kg). Transplanted scaffolds in Group 1 and 2 were evaluated after euthanasia 14 days (*n* = 7/group), 30 days (*n* = 6/group), and 120 days (Group 1, *n* = 6; Group 2, *n* = 5) after transplantation. The spleen from every animal at every time point was also explanted and weighed.

### Immunohistochemistry and immune cell infiltration quantification

To quantify the infiltration of immune cells into the graft at each time point, a large segment was excised from the center of each graft and this biopsy was fixed in 4% paraformaldehyde (Histolab, Gothenburg, Sweden). A uterus biopsy was also collected from six naïve rats. Each specimen was then dehydrated and embedded in paraffin blocks. Each specimen was cut in 5 μm sections and mounted on microscope slides. Hematoxylin and eosin staining was conducted according to standard protocols. Immunohistochemistry was conducted after an antigen retrieval step (pressure cooking in citric acid, pH = 6.0) using the biotin-free alkaline phosphatase Mach 3 staining kit and the subsequent vulcan fast red kit according to the manufacturer’s instructions (Biocare Medical, Pacheco, CA, USA). The primary antibodies used were diluted in tris-buffered saline with 0.2% Tween 20 and incubated for 1 h at room temperature. All used antibodies came from Abcam (Cambridge, UK) and included; anti-CD4 (T cells; ab33775 1:100), anti-CD8-α (cytotoxic T cells; ab33786 1:1000), anti-FOXP3 (T_Regs_; ab75763; 1:500) anti-CD22 (B cells; ab197650 1:200), anti-CD68 (pan-macrophage; ab125212 1:1000), anti-CD86 (class M1 macrophage; ab220188; 1:100), anti-CD163 (class M2 macrophage; ab182422 1:200). The stained sections were mounted in DPX after a dehydration step. The total number of positively stained cells in six random regions (each region = 0.074 mm^2^) of the endometrium and myometrium, respectively, from two to three separate sections per graft were quantified blinded in a microscope at 400× magnification. Immunofluorescent staining was conducted using primary antibodies for anti-e-cadherin (epithelial cells; 1:200; ab76055; Abcam), anti-smooth muscle cell actin (SMA, muscle cells;1:300; ab5694; Abcam), or anti-GFP (1:300; ab290; Abcam) for 1 h at room temperature. CY3 or an Alexa Fluor 488 secondary antibody was used for 1 h at room temperature (Thermo Fisher; 1:300). Sections were then washed in PBS, and subsequently cover slipped with Dako fluorescent mounting media.

### Blood samples and plasma analysis

Blood samples were taken from the transplanted animals’ lateral tail vein using a butterfly catheter (Sarstedt, Helsingborg, Sweden) on day 3, 7, 14, 30, and 120 post transplantation. Blood was also collected from 12 naïve rats for base line values. The blood was aspirated into a collection tube coated with (EDTA; Sarstedt) and kept on ice until centrifugation at 500*g* for 10 min at 4°C. The plasma was collected and stored at −20°C until the cytokine profile was analyzed using the FirePlex®−96 inflammation (rat) immunoassay panel (ab235665; Abcam). The analysis was performed by Abcam and included the following 16 proteins; C-X-C motif chemokine ligand 1 (CXCL1), granulocyte-macrophage colony-stimulating factor (GM-CFS), interferon γ (IFNγ), interleukin (IL) 1β, IL-2, IL-4, IL-5, IL-9, IL-10, IL-12p70, IL-13, IL-17A, monocyte chemoattractant protein 1 (MCP1), macrophage inflammatory protein (MIP) 1α, MIP-1β, and tumor necrosis factor α (TNFα). Numbers of sample for each group and time point can be viewed in Table 1 and was dependent on the technical success of taking blood samples from each animal at each specific time points (Table 1).

**Table 1.** Number of samples measured in the FirePlex^®^ analysis per group and time point.

FirePlex^®^ analysis	Day 3	Day 7	Day 14	Day 30	Day 120
Group 1 (scaffold with MSCs)	*n* = 14	*n* = 14	*n* = 14	*n* = 4	*n* = 6
Group 2 (scaffold without cells)	*n* = 20	*n* = 13	*n* = 13	*n* = 6	*n* = 4
Naïve rat plasma (*n* = 12)					

### Statistical analysis

All data were tested for normal distribution using the Shapiro-Wilk test in the GraphPad Prism 9 software (GraphPad, CA, USA). Parametric data were assessed with Welch’s *t*-test for two group comparison. If more than two normally distributed groups were compared, the one-way ANOVA with Tukey’s corrections was used. For non-parametric data, the Mann-Whitney *U*-test was used for two group comparison, or the Kruskal-Wallis test with Dunn’s post-hoc test for multiple group comparison.

## Results

### Macroscopic observations of the grafts

No macroscopic discrepancies were observed between the acellular or recellularized grafts at any time point during tissue harvesting. Graft revascularization was observed as early as 14 days after engraftment (Figure 1(E)). Some animals experienced graft adhesion to the surrounding omentum and intestinal tissue, and a few operated uteri were affected by stenosis at the anastomosis site with a subsequent buildup of fluids proximally to the lesion site. However, the graft shape was well maintained during the first 30 days with no obvious signs of lumen collapse (Figure 1(F)). At 120 days after transplantation, more adhesions to the surrounding tissue were observed. Several grafts seemed to have lost their physical structure and had reduced in size (Figure 1(G)). Several grafts appeared to have a collapsed or reduced luminal diameter compared to grafts observed at the earlier time points. The weight of the spleens isolated from each animal remained around 1 g throughout the experiment, independent of graft type and time point (Supplemental Figure 1).

### Microscopic observations of the grafts

**Figure 2.**
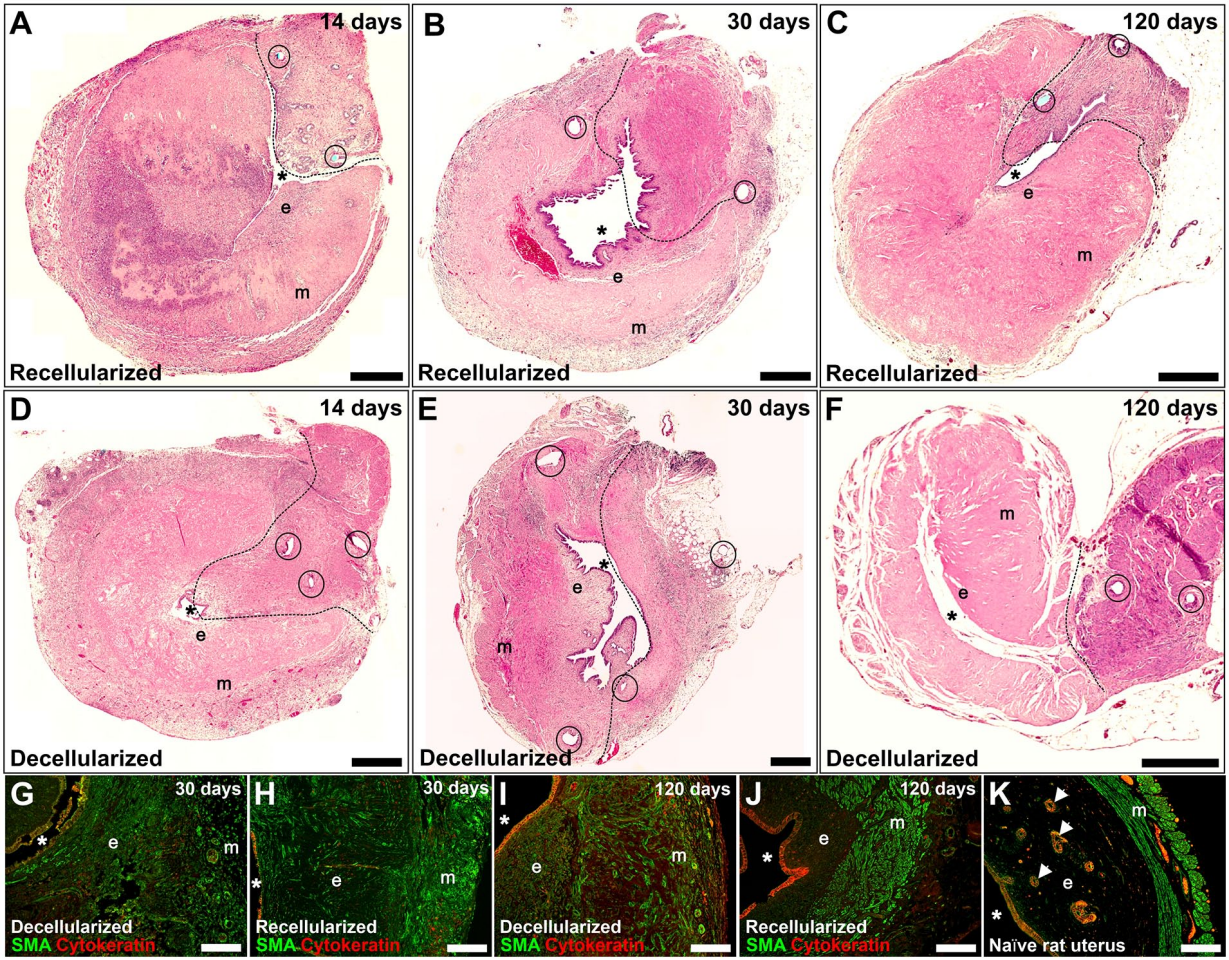
Hematoxylin and eosin-stained representative sections from acellular and recellularized grafts obtained at 14, 30, and 120 days, respectively (A–F). Grafts previously recellularized with MSCs showed improved myometrium organization compared with acellular grafts, indicated by the smooth muscle cell actin staining (green; (G–K)). However, endometrial glandular tissue was not well reformed in any grafts, indicated by the cytokeratin staining (red; (G–K)). Circles indicate suture sites, dotted lines represents the graft-native tissue junction (A–F). Scale bars (A–F) = 500 µm; (G–K) = 200 µm. m: myometrium; e: endometrium; *: uterus luminal space.

Hematoxylin and eosin staining confirmed that the decellularization was successful (Figure 1(B)). Stained sections of recellularized segments showed that the added GFP^+^ MSCs had predominantly localized to the superficial layers of the scaffolds and in isolated pockets near the injection sites after 2 weeks in vitro (Figure 1(C)). At the first time point (14 days after engraftment), no GFP^+^ MSCs were found (stained sections were negative for GFP; Supplemental Figure 1) while there was a large population of infiltrated cells (Figure 2(A) and (D)). No major differences were observed between the experimental groups in the endometrial compartments of the scaffolds after 30 days (Figure 2(G) and (H)). After 120 days, the endometrial layer seemed better organized in recellularized grafts (Figure 2(I) and (J)). The luminal epithelial layer regenerated well in both graft types, however, few glandular structures were found in the recipients, independent of graft type. The myometrium layer was clearly better regenerated in recipients of the recellularized grafts at both 30 and 120 days after engraftment (Figure 2(G)–(K)).

### Immune modulation during the first 14 days

**Figure 3.**
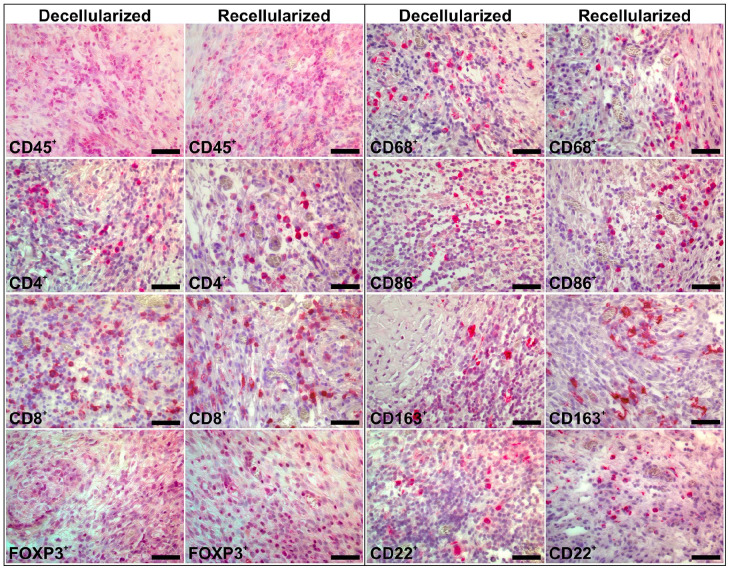
Representative images of the biotin-free alkaline phosphatase Mach 3 immunohistochemistry stained sections taken from the myometrial compartment of the decellularized or recellularized grafts, respectively, 2 weeks after transplantation. Positively stained cells (red) included CD45^+^ leukocytes, CD4^+^ T cells, CD8^+^ cytotoxic T cells, FOXP3^+^ T_Regs_, CD68^+^ pan-macrophages, CD86^+^ class M1 macrophages, CD163^+^ class M2 macrophages, and CD22^+^ B cells. Scale bars = 50 µm.

**Figure 4.**
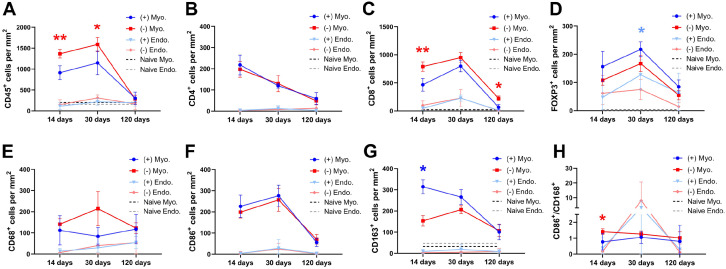
The mean values (±SEM) of the quantified cells that had infiltrated the graft at the three different time points after engraftment. MSCs-recellularized grafts (+; blue); acellular grafts (−; red); Myo: myometrial compartment; Endo: endometrial compartment; CD45^+^ leukocytes (A); CD4^+^ T cells (B); CD8^+^ cytotoxic T cells (C); FOXP3^+^ T_Regs_ (D); CD68^+^ pan-macrophages (E); CD86^+^ class M1 macrophages (F); CD163^+^ class M2 macrophages (G), and the proportion of class M1 macrophagesclass and M2 macrophages (H). Dotted lines are the mean value of naïve uterus tissue.

Cell counts 2 weeks after transplantation showed that there was significantly fewer infiltrating CD45^+^ leukocytes in the grafts containing MSCs which predominantly were localized to the myometrium compartment of the scaffolds (Figures 3 and 4). There was also a lower population of CD8^+^ cytotoxic T cells, and a higher population of resident CD163^+^ class M2 macrophages in the myometrial compartment of the recellularized grafts (Figure 4(G)). Furthermore, there was a significantly lower ratio for the inflammatory-related CD86^+^ class M1 versus the regeneration-related CD163^+^ class M2 macrophages in recellularized grafts (Figure 4(H)). The presence of MSCs did not affect the number of infiltrating CD4^+^ T cells, FOXP3^+^ T_Reg_ cells, or infiltrated CD68^+^ macrophages, CD86^+^ class M1 macrophages (Figure 4) or CD22^+^ B cells (Supplemental Figure 2) 14 days after transplantation. The mean cell density of respective immune cell type was additionally quantified in sections from naïve rat myometrium and endometrium (Figure 4; Supplemental Figure 1(B)).

**Figure 5.**
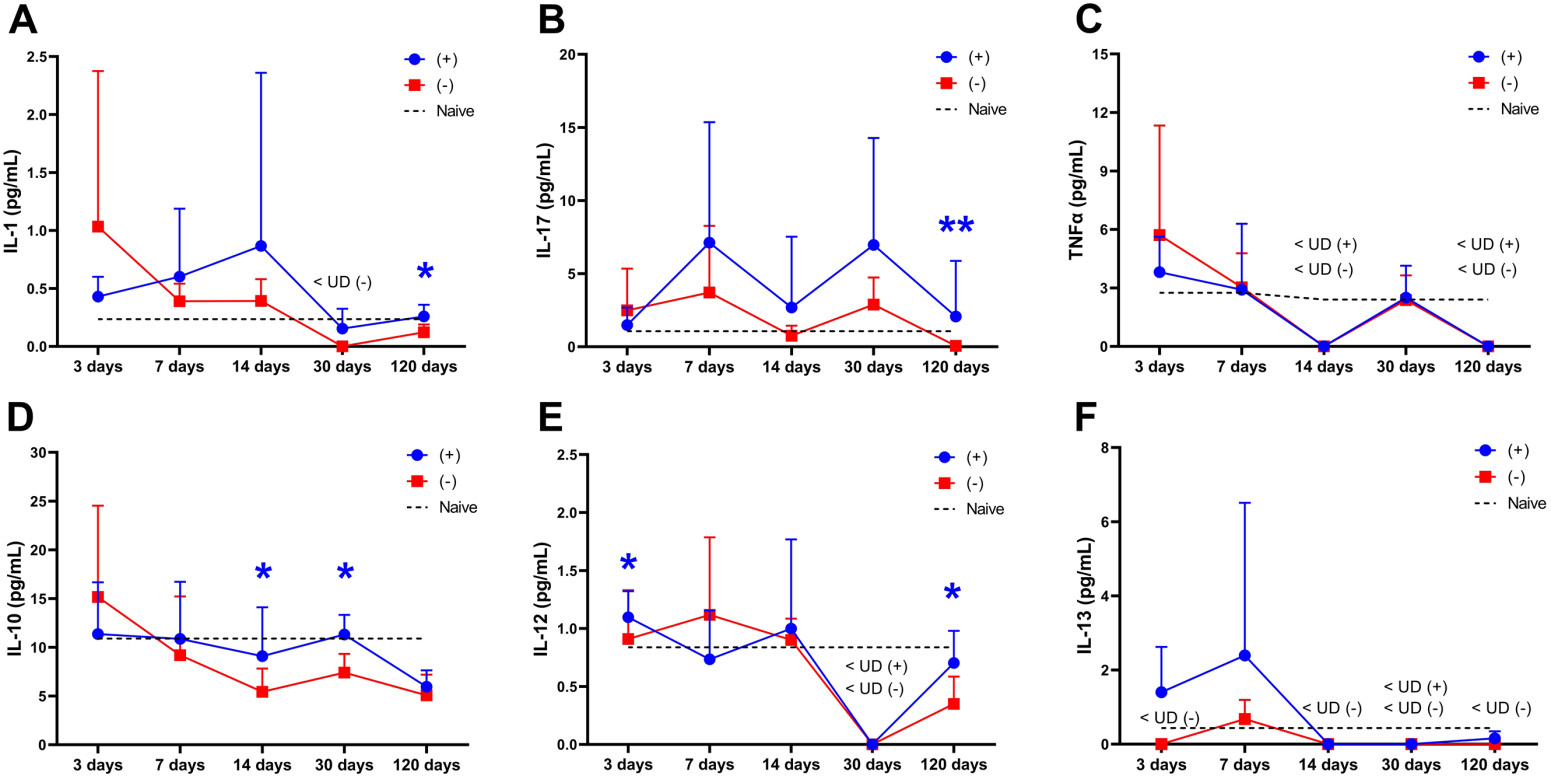
Pro-inflammatory (A–C) and anti-inflammatory (D–F) cytokine plasma levels from recipients of MSCs-recellularized grafts (+; blue) and acellular grafts (−; red) were quantified at five different time points throughout the study. Dotted lines represent the mean value in naïve rat blood plasma. Mean ± SD. *p < 0.05; **p < 0.01.

The plasma levels for the pro-inflammatory cytokines IL-1, IL-17, and TNFα were not significantly different between the acellular and the recellularized grafts during the first 14 days (Figure 5(A)–(C)). The levels of the anti-inflammatory cytokine IL-10 were similar between the groups during the first week, but grafts recellularized with MSCs resulted in higher IL-10 plasma levels on day 14 (Figure 5(D)). IL-12 was significantly higher in recipients of recellularized grafts compared to recipients of acellular grafts on day 3 (Figure 5(E)), but levels then became similar between the groups over time. IL-13 was also higher in the animals with recellularized scaffolds at the earliest time point compared to the undetectable plasma concentration in recipients of acellular grafts (Figure 5(F)). The levels of IL-13 then increased in both groups on day 7 but subsequently dropped to very low concentrations.

**Figure 6.**
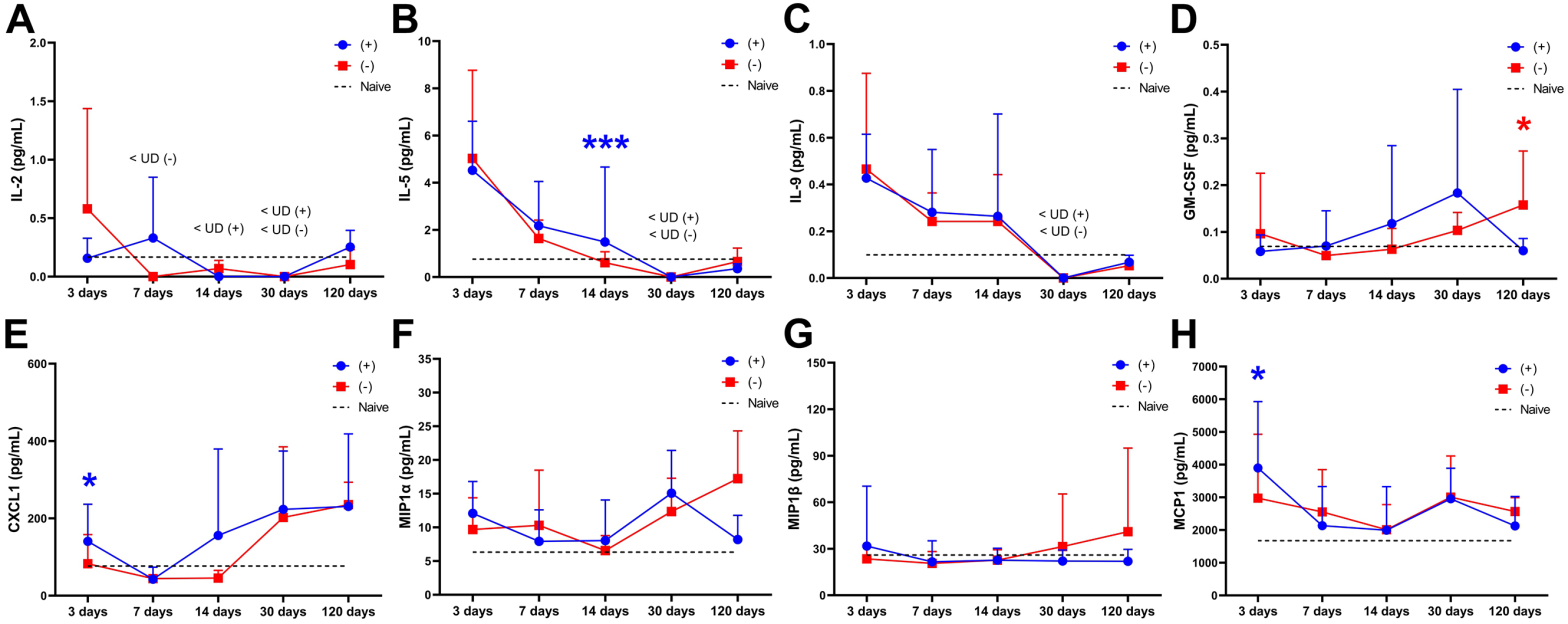
Cytokine plasma levels associated with the adaptive immune response (A–D) and various chemokines (E–H) from recipients of MSCs-recellularized grafts (+; blue) and acellular grafts (−; red) were quantified at five different time points throughout the study. Dotted lines represent the mean value in naïve rat blood plasma. Mean ± SD. *p < 0.05; ***p < 0.001.

The measured levels of the cytokines IL-2, IL-9, and CM-CSF, which are associated with the adaptive immune response, remained similar between the experimental groups during the first 14 days. However, IL-5 did not decline as rapidly as the acellular group on day 14, and a higher level of IL-5 was detected in recellularized grafts (Figure 6(A)–(D)). Initially, the plasma levels of the chemokines CXCL1 and MCP1 were higher in recipients of recellularized grafts on day 3 but the levels became similar between the groups after that. Plasma levels were also similar between the groups for the chemokines MIP1α or MIP1β (Figure 6(E)–(H)). The cytokine levels in naïve rat blood plasma were also measured and were included in the graphs for base line values (Figures 5 and 6)

### Immune modulation 30 days post transplantation

There was a significantly higher number of infiltrated CD45^+^ leukocytes in the myometrial compartment of the acellular grafts on day 30 post engraftment compared to MSC-recellularized grafts (Figure 4(A)). The number of T cells was similar between the groups except for the higher number of FOXP3^+^ T_Regs_ found in the endometrial compartment of recellularized grafts (Figure 4(B)–(D)). There was no difference in the macrophage populations between the groups at this time point, or in the M1/M2 ratio (Figure 4(E)–(H)) or in the CD22^+^ B cell population (Supplemental Figure 2).

Plasma levels for the pro-inflammatory cytokines IL-1, TNFα, and IL-17 were also similar between the experimental groups (Figure 5(A)–(C)). The levels of the anti-inflammatory cytokine IL-12 and IL-13 were too low to be detected in any grafted animals on day 30. However, a significantly higher IL-10 level was detected in recellularized grafts compared to acellular grafts that showed declining levels (Figure 5(D)–(F)). There were undetectable levels of the adaptive response-related cytokines IL-2, IL-5, and IL-9 on day 30, and the detectable GM-CSF level was similar between the grafts. The examined chemokines were also similar between the groups (Figure 6).

### Immune modulation 120 days post transplantation

At this late time point after engraftment, we noticed a general decline in CD45^+^ leukocyte presence within the grafts of both groups, in particular in the myometrial compartment of the grafts. The general decline included all sub types of T cells (Figure 4(A)–(D)) and B cells (Supplemental Figure 2). However, there was still significantly more CD8^+^ cytotoxic T cells in the acellular grafts. There were no significant differences between the groups concerning the number of resident CD68^+^, CD86^+^, or CD163^+^ macrophages (Figure 4(E)–(H)).

Plasma levels for the pro-inflammatory cytokines IL-1 and IL-17 were significantly higher in recipients of the recellularized graft compared with recipients of the acellular graft (Figure 5(A) and (B)). TNFα levels were undetectable at this time point. The anti-inflammatory cytokine levels of IL-10 and IL-13 remained similar between the groups, but IL-12 plasma levels were significantly higher in recipients of the recellularized graft compared with recipients of the acellular graft (Figure 5(D)–(F)). Apart from the GM-CSF plasma level, which were higher in acellular recipients, the adaptive response-related cytokines IL-2, IL-5, and IL-9 were similar between the groups, and the chemokine levels for CXCL1, MIP1α, MIP1β, and MCP1 also remained statistically comparable at this late time point (Figure 6).

## Discussion

The immune-modulating effects from MSCs have been well studied in a number of different injury models but their effects have not been sufficiently studied in combination with decellularized tissue transplantation. The results presented herein suggest that the MSCs used to recellularize rat uterus scaffolds contributed to a reduction of infiltrated leukocytes during the first month after engraftment, and skewed the immune system toward a regenerative faith by reducing the CD8^+^ cytotoxic T cell population, increasing the presence of FOXP3^+^ T_Regs_ and CD163^+^ class M2 macrophages. Additionally, these events were accompanied by higher anti-inflammatory plasma levels for IL-10, IL-12, and IL-13 in recipients of recellularized grafts compared with recipients to acellular grafts.

Neutrophils and mast cells are rapid leukocyte responders following tissue engraftment and are usually associated to acute inflammation triggered by DAMPs and potential pathogens. They also secrete cytokines and growth factors to recruit additional inflammatory cells such as phagocytizing monocytes and macrophages. While neutrophils have been associated to contribute to the resolution of inflammation^20^ and stimulate macrophage M2 polarization after myocardial infarction,^21^ mast cells have been linked to activities related to inflammation and scarring formation. For example, mast cells effectively recruit tissue destructive eosinophils and are a main contributing source to the secondary inflammatory injury phase and scarring formation after traumatic brain injury.^22^ They have also been shown to persist at high numbers in chronic wounds.^23^ Hence, it would have been valuable to quantify any potential infiltration differences of these specific leukocytes to investigate if the MSCs modulated these events. However, neutrophils, mast cells or eosinophils usually infiltrate transplanted tissue within hours after implantation, and thus, would require a much earlier experimental time point than our first examination (14 days after engraftment). Nevertheless, the total number of leukocytes were reduced in grafts that had been recellularized with MSCs. In addition to this observation, we detected higher plasma levels for the anti-inflammatory cytokines IL-10, IL-12, and IL-13 in recipients of MSCs recellularized grafts. IL-10 is expressed by several immune cells from both the innate and the adaptive response, including neutrophils, mast cells, T cells and macrophages^24^ and can moderate the release of pro-inflammatory cytokines and was shown to be important for the generation and maintenance of the CD4^+^ T cell population.^24^ T cells have dynamic functions that depends on the variety of cytokines produced. These cytokines can either stimulate or inhibit tissue regeneration. For example, there is evidence that CD4^+^ and CD8^+^ T cells inhibited bone regeneration in humans, and that the healing was accelerated when CD8^+^ T cells were depleted.^25^ It was also shown that T cells could inhibit MSCs-driven bone formation via IFNγ and TNFα expression in a mouse model.^26^ In our study, we did not detect a significant rise in the systemic pro-inflammatory cytokine levels for IL-1, IL-17, or TNFα. However, we found almost twice as many CD8^+^ cytotoxic T cells in acellular grafts 14 days after transplantation compared with grafts that were recellularized with MSCs. Interestingly, this ratio difference lasted throughout the 120 days long experiment, even if MSCs are known to be removed from similar uterus grafts within days,^9^ which was also confirmed herein, and in a recently published article that evaluated the MSC survival after either a subcutaneous, intrapancreatic or intrasplenic transplantation in a mouse model.^27^ However, the immune modulating events from MSCs were shown to persist even through metabolically inactivated, apoptotic or fragmented MSCs and might explain our long-term advantageous immune modulating results in the recellularized scaffolds herein.^28,29^ Hence, our data support the “dying stem cell hypothesis” that apoptotic MSCs modulate the innate and adaptive immune response.^30^ We further believe that these beneficial effects can be improved by establishing higher recellularization efficiencies in the decellularized scaffolds since the MSCs immunosuppressive effects on CD4^+^ and CD8^+^ T cell proliferation, and their stimulation for T_Regs_ differentiation are dose-dependent.^31^ In our study, the recellularization was mostly localized to the superficial layers of the scaffolds and to some confined high cell density areas around the injection sites. This is a common major hurdle for decellularized tissues that undergo recellularization in vitro, independent of scaffold origin. However, the successfully recellularized external surface of the grafts used herein may have acted as a physical and bioactive barrier for infiltrating leukocytes that effectively modulated inflammation.

Furthermore, we found more FOXP3^+^ T_Regs_ in the recellularized grafts, in particular after 30 days. This subgroup of T cells has shown to be critical for the repair and regeneration of many tissues, including bone, skin and muscle.^32^ FOXP3^+^ T_Regs_ are also responsible for an increased production of anti-inflammatory cytokines, including IL-10, that favor tissue repair and the polarization of macrophages toward a class CD163^+^ M2 phenotype.^33^ The cell counts made from the pan-macrophage marker CD68 did not reveal any group differences at any time point in our study, and the number of infiltrating CD86^+^ class M1 macrophages was also similar between the groups. However, there was a significantly larger CD163^+^ class M2 macrophage population in grafts that were recellularized with MSCs during the first month after transplantation, suggesting that MSCs further potentiate the immune system toward a regenerative stage together with the decellularized scaffolds.

During the healing process, the luminal epithelial cells seemed to have migrated horizontally along the epithelial architecture to cover the luminal surface area of the graft. This correlates to earlier described observations after decellularized uterus tissue transplantation in the mouse and rat,^10,34^ and after a biodegradable polymer scaffold transplantation in the rabbit.^35^ We speculate that a similar process might occur in the endometrial and myometrial compartments, and that the MSCs in recellularized scaffolds potentiated this process, even after the MSCs disappearance. It has, for example, been shown that condition media and extracellular vesicles from MSCs accelerate the recovery and contractile function of muscle tissue in a rat model and further stimulated new muscle fiber formation, modulated inflammation, fibrosis, and myogenesis mechanisms.^36^ A similar treatment also showed to polarize macrophage phenotypes, alleviate the inflammatory events, and stimulate muscle regeneration.^37^ The immunomodulation seen in our study might explain the improved myometrial regeneration in the scaffolds previously recellularized with MSCs. However, the low glandular reformation in our grafts was unexpected. Studies using smaller uterus grafts reported successful recovery of this tissue type.^10,34^ It is still ambiguous how these glandular structures regenerate, but results from another study suggest that they might be formed by recruited host cells of stroma cell origin, or from a source of endometrial stem/progenitor cells or from a glandular progeny.^10,38^ It is likely that this process is further dependent of the scaffold’s in vivo degradation properties. The grafts used in the study described herein were of a significantly larger size compared to previously assessed scaffolds in the literature, which could influence the glandular reformation. Hence, from a regenerative perspective, scaffold modifications that prolongs the degradation time may be needed to meet the overall objective; to create a bioengineered transplantable graft suitable for fertility-restoring surgery of uterine injuries caused for example, from myomectomy, adenomyomectomy or trachelectomy after uterine cervical cancer, and/or to reduce the life-threatening risk of uterine rupture during pregnancy. Furthermore, the use of primary uterine cells, and/or uterine progenitor/stem cell-like cells may prove essential for a complete uterus regeneration and should thus also be considered for the recellularization process,^39–45^ perhaps as a combination with MSCs.

Lastly, we speculate that the sequence of events after transplantation of the recellularized grafts in our study included an immediate inflammatory response following the engraftment with raised plasma levels for the chemokines CXCL1 and MCP1. However, sometime after the first and second week, the MSCs recellularized grafts modulated the adaptive immune system better than the acellular grafts through an increase in IL-10 plasma levels, either directly or indirectly via FOXP3^+^ and/or CD163^+^ M2 macrophages, and simultaneously reduced the presence of CD8^+^ cytotoxic T cells. This is consistent with previous observations when MSCs were infused intravenously in a mouse model that resulted in a similar but faster inflammatory systemic response with increased plasma levels of IL-6, CXCL1 and MCP1 a few hours after the infusion.^46^ In the same study, the subsequent accumulated MSCs and macrophage aggregation in the recipients’ lung tissue then reduced the inflammatory response after 3 days. It is plausible that the observed regeneration-favorable effects from the MSCs in our study were delayed and prolonged due to their embedment in the decellularized scaffold compared with the direct intravenous cell infusion approach. While the detailed mechanisms behind the MSCs immune modulating functions and their regenerating effects still remain ambiguous, our study clearly showed that MSCs can serve an important therapeutic role to create a pro-regenerative immune microenvironment together with decellularized tissue transplantation.

## Supplemental Material

sj-docx-1-tej-10.1177_20417314221118858 – Supplemental material for Mesenchymal stem cells establish a pro-regenerative immune milieu after decellularized rat uterus tissue transplantation Click here for additional data file. Supplemental material, sj-docx-1-tej-10.1177_20417314221118858 for Mesenchymal stem cells establish a pro-regenerative immune milieu after decellularized rat uterus tissue transplantation by Edina Sehic, Emy Thorén, Ingigerdur Gudmundsdottir, Mihai Oltean, Mats Brännström and Mats Hellström in Journal of Tissue Engineering

sj-jpg-2-tej-10.1177_20417314221118858 – Supplemental material for Mesenchymal stem cells establish a pro-regenerative immune milieu after decellularized rat uterus tissue transplantation Click here for additional data file. Supplemental material, sj-jpg-2-tej-10.1177_20417314221118858 for Mesenchymal stem cells establish a pro-regenerative immune milieu after decellularized rat uterus tissue transplantation by Edina Sehic, Emy Thorén, Ingigerdur Gudmundsdottir, Mihai Oltean, Mats Brännström and Mats Hellström in Journal of Tissue Engineering

sj-jpg-3-tej-10.1177_20417314221118858 – Supplemental material for Mesenchymal stem cells establish a pro-regenerative immune milieu after decellularized rat uterus tissue transplantation Click here for additional data file. Supplemental material, sj-jpg-3-tej-10.1177_20417314221118858 for Mesenchymal stem cells establish a pro-regenerative immune milieu after decellularized rat uterus tissue transplantation by Edina Sehic, Emy Thorén, Ingigerdur Gudmundsdottir, Mihai Oltean, Mats Brännström and Mats Hellström in Journal of Tissue Engineering
