# Supplementary material for: Mesenchymal stem cells establish a pro-regenerative immune milieu after decellularized rat uterus tissue transplantation
Source: J Tissue Eng. 2022 Aug 20;13:20417314221118858. doi: 10.1177/20417314221118858 (PMC9393937; doi:10.1177/20417314221118858)
Supplement: sj-docx-1-tej-10.1177_20417314221118858 – Supplemental material for Mesenchymal stem cells establish a pro-regenerative immune milieu after decellularized rat uterus tissue transplantation [file sj-docx-1-tej-10.1177_20417314221118858.docx]

**
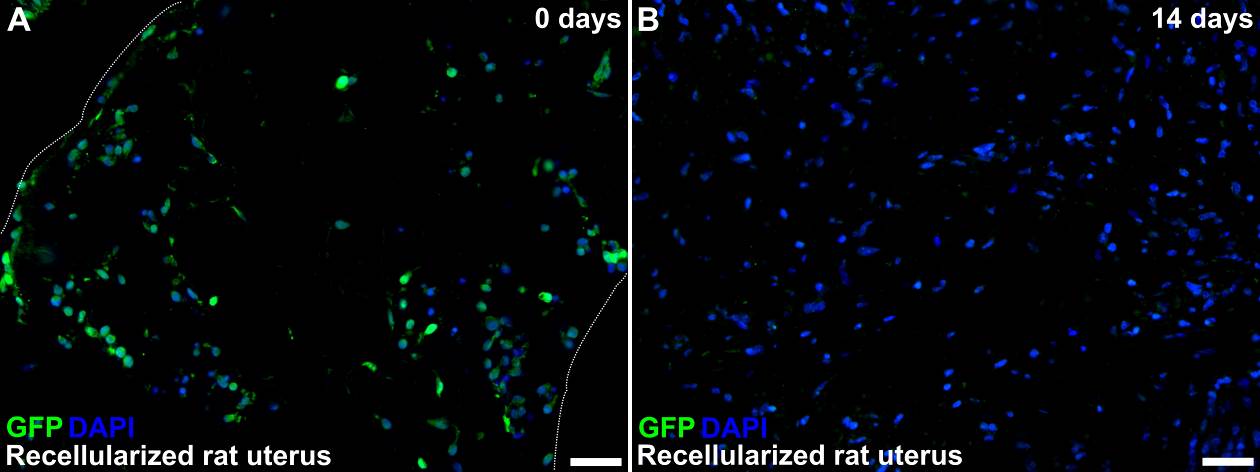
**

**Supplementary figure 1**

Green fluorescent protein (GFP) labelled mesenchymal stem cells were clearly stained positive in grafts at the time of transplantation (0 days; A). No GFP labeled cells were visualized in the grafts 14 days after transplantation (B). Scale bars = 100µm. Scaffold area indicated by the dotted lines (A).

**
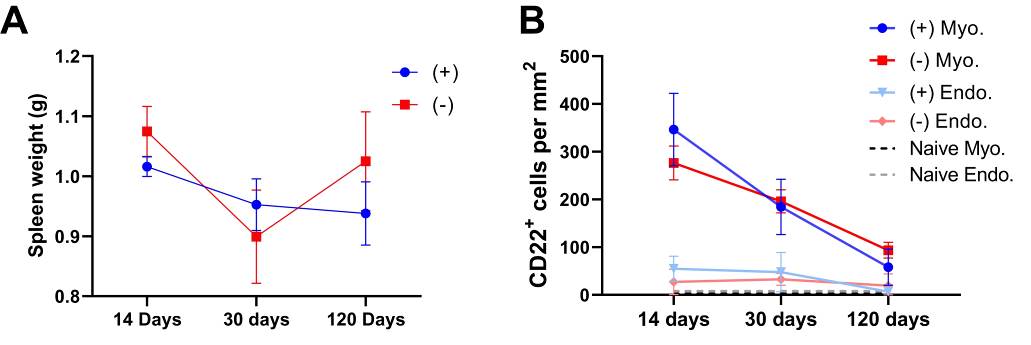
**

**Supplementary figure 2.** The weight of the spleen from each recipient was similar between the groups (A), and the number of quantified CD22^+^ B cells were also comparable between the groups. Mean values ±SEM; MSCs-recellularized grafts (+; blue); acellular grafts (-; red); Myo, myometrial compartment, Endo, endometrial compartment.
