# Supplementary figures and images for: Mesenchymal stem cells establish a pro-regenerative immune milieu after decellularized rat uterus tissue transplantation
Source: J Tissue Eng. 2022 Aug 20;13:20417314221118858. doi: 10.1177/20417314221118858 (PMC9393937; doi:10.1177/20417314221118858)

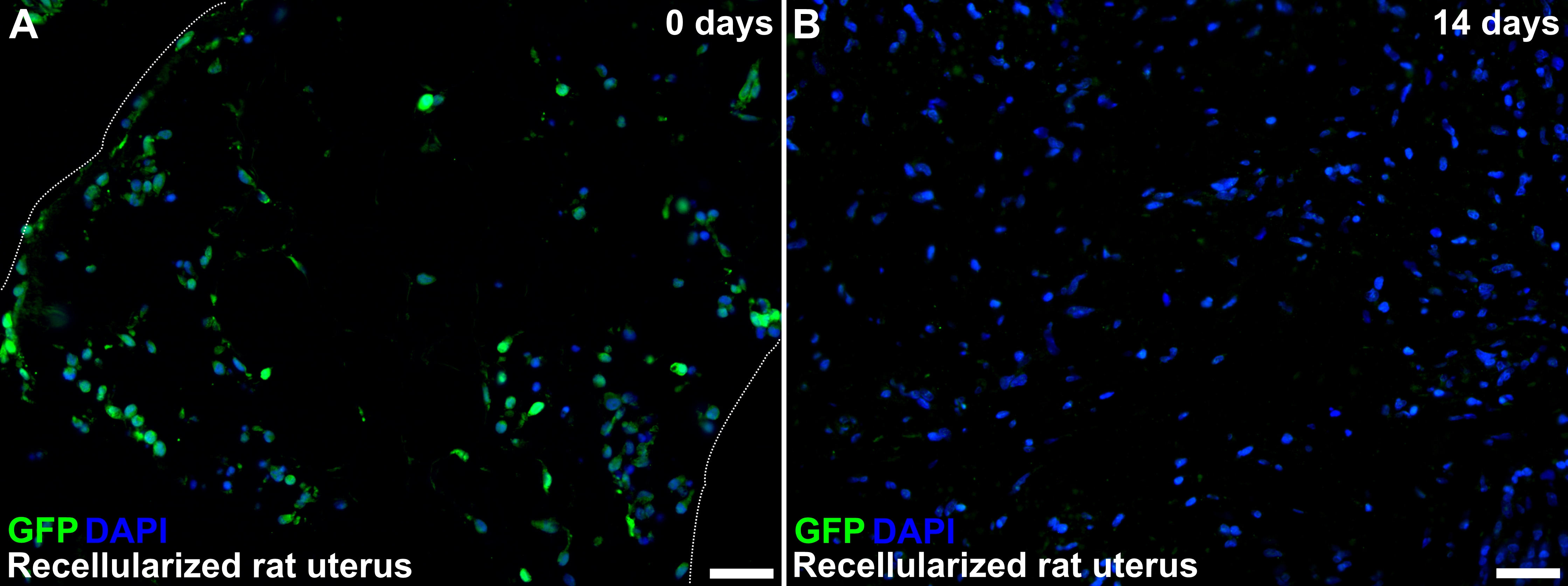

Supplement: sj-jpg-2-tej-10.1177_20417314221118858 – Supplemental material for Mesenchymal stem cells establish a pro-regenerative immune milieu after decellularized rat uterus tissue transplantation [file sj-jpg-2-tej-10.1177_20417314221118858.jpg]

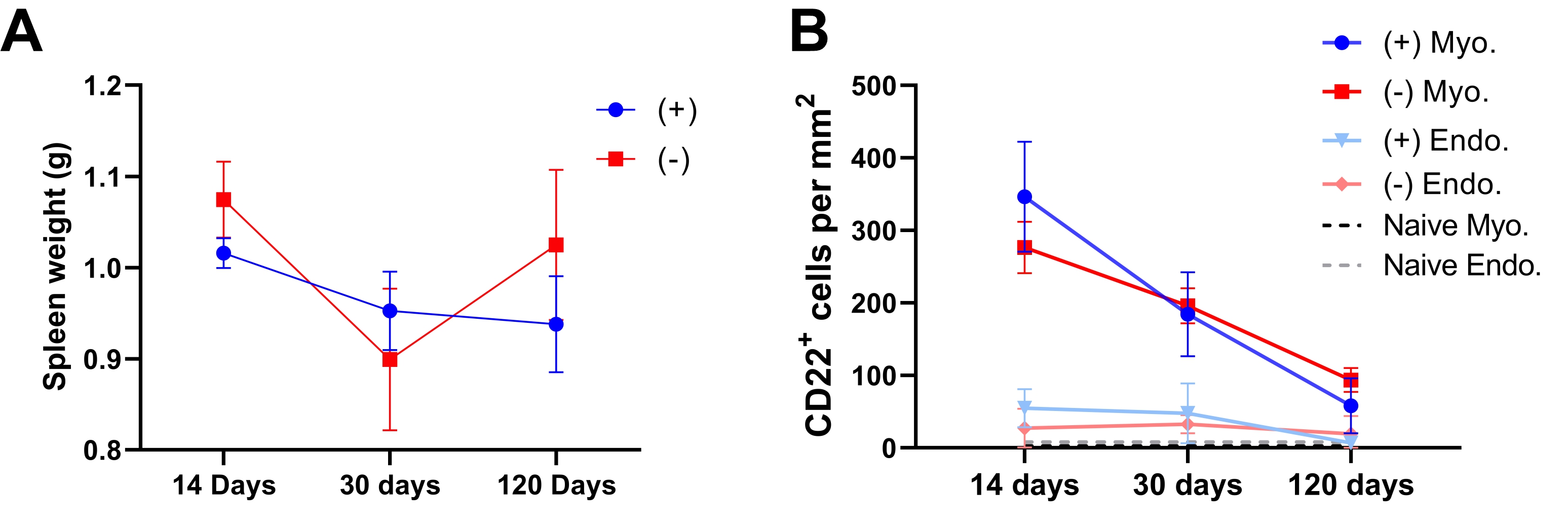

Supplement: sj-jpg-3-tej-10.1177_20417314221118858 – Supplemental material for Mesenchymal stem cells establish a pro-regenerative immune milieu after decellularized rat uterus tissue transplantation [file sj-jpg-3-tej-10.1177_20417314221118858.jpg]
